# Quantitative Dissection of the Proximal *Ciona brachyury* Enhancer

**DOI:** 10.3389/fcell.2021.804032

**Published:** 2022-01-21

**Authors:** Kotaro Shimai, Michael Veeman

**Affiliations:** Division of Biology, Kansas State University, Manhattan, KS, United States

**Keywords:** Ciona, tunicate, enhancer, brachyury, cis-regulatory analysis, gene regulatory networks, transcriptional regulation

## Abstract

A major goal in biology is to understand the rules by which cis-regulatory sequences control spatially and temporally precise expression patterns. Here we present a systematic dissection of the proximal enhancer for the notochord-specific transcription factor *brachyury* in the ascidian chordate *Ciona*. The study uses a quantitative image-based reporter assay that incorporates a dual-reporter strategy to control for variable electroporation efficiency. We identified and mutated multiple predicted transcription factor binding sites of interest based on statistical matches to the JASPAR binding motif database. Most sites (Zic, Ets, FoxA, RBPJ) were selected based on prior knowledge of cell fate specification in both the primary and secondary notochord. We also mutated predicted Brachyury sites to investigate potential autoregulation as well as Fos/Jun (AP1) sites that had very strong matches to JASPAR. Our goal was to quantitatively define the relative importance of these different sites, to explore the importance of predicted high-affinity versus low-affinity motifs, and to attempt to design mutant enhancers that were specifically expressed in only the primary or secondary notochord lineages. We found that the mutation of all predicted high-affinity sites for Zic, FoxA or Ets led to quantifiably distinct effects. The FoxA construct caused a severe loss of reporter expression whereas the Ets construct had little effect. A strong Ets phenotype was only seen when much lower-scoring binding sites were also mutated. This supports the enhancer suboptimization hypothesis proposed by Farley and Levine but suggests that it may only apply to some but not all transcription factor families. We quantified reporter expression separately in the two notochord lineages with the expectation that Ets mutations and RBPJ mutations would have distinct effects given that primary notochord is induced by Ets-mediated FGF signaling whereas secondary notochord is induced by RBPJ/Su(H)-mediated Notch/Delta signaling. We found, however, that ETS mutations affected primary and secondary notochord expression relatively equally and that RBPJ mutations were only moderately more severe in their effect on secondary versus primary notochord. Our results point to the promise of quantitative reporter assays for understanding cis-regulatory logic but also highlight the challenge of arbitrary statistical thresholds for predicting potentially important sites.

## Introduction

The small, simple chordate embryo, invariant cleavage patterns, compact genome and ease of transgenesis in *Ciona* and other ascidians have made them important models for the systematic dissection of developmental Gene Regulatory Networks (GRNs) (Imai et al., 2006; Satoh, 2014; Shi et al., 2005; Kubo et al., 2009; Satou and Peter, 2020). There are robust methods for identifying transcription factors and signaling molecules of interest in distinct cell types, perturbing their functions, and identifying downstream genes that are differentially expressed in response to those perturbations (Satou et al., 2001a; Satou et al., 2001b; Satou et al., 2003; Imai et al., 2004; Sasaki et al., 2014; Stolfi et al., 2014). With the advent of single cell RNAseq and cell-type specific CRISPR gene disruption, these methods are becoming quite powerful (Stolfi et al., 2014; Gandhi et al., 2017; Cao et al., 2019; Zhang et al., 2020; Winkley et al., 2021). As in other model organisms, however, one of the biggest challenges in GRN analysis is determining whether transcriptional regulatory effects are direct or indirect. Some ChIPchip and ChIPseq data are available in *Ciona* (Kubo et al., 2010; Oda-Ishii et al., 2016; Tokuhiro et al., 2017), but TF binding to particular regulatory regions does not necessarily indicate that it is functionally important (Yang et al., 2006; Hu et al., 2007; Biggin, 2011). The gold standard for determining whether transcriptional interactions are direct or indirect usually involves cis-regulatory analysis (Stolfi and Christiaen, 2012; Irvine, 2013). If the mutation of known or predicted Transcription Factor Binding Sites (TFBS) in an enhancer construct abrogates reporter expression in the expected cell type(s), it provides strong though not unequivocal support for the interaction being direct.

*Ciona* is particularly well suited to fast-paced cis-regulatory analysis because of the unusual ability to easily electroporate reporter constructs into large numbers of fertilized eggs. Transcriptional reporter assays have been a mainstay of the ascidian research community since Corbo and colleagues first implemented *Ciona* egg electroporation nearly 25 years ago (Corbo et al., 1997). There has been considerable progress since then in terms of new electroporation protocols (Zeller et al., 2006; Vierra and Irvine, 2012; Zeller, 2018), more refined and adaptable vector systems (Roure et al., 2007), and a shift away from LacZ as the reporter of choice towards a broad range of fluorescent protein variants and other reporters.

Despite the widespread use of electroporated reporter assays in *Ciona*, there are several interrelated issues that complicate cis-regulatory analysis. One is that reporter expression is difficult to quantify. Expression patterns are frequently assessed in a purely qualitative framework, or else they are crudely quantified in term of the fraction of embryos with any detectable transgene expression. A related issue is that there is variability in transfection efficiency between different electroporations that is difficult to control for. This may not be a major concern if a particular mutation eliminates reporter expression, but it is problematic when assessing subtler quantitative effects. In other contexts it has become common to use a dual reporter strategy to control for transfection efficiency (Dyer et al., 2000; McNabb et al., 2005). In transient transfection assays in mammalian cell lines, for example, firefly and *Renilla* luciferase are widely used as orthogonal reporters. Sensitive, high dynamic range luciferase assays can be used to independently quantify both reporters. One is typically driven by a ubiquitously expressed cis-regulatory module that can be used as a control for transfection efficiency, and the other by a regulated cis-regulatory module of interest. Dividing the quantitative reporter value for the regulated CRM of interest by the value for the control reporter provides a normalized value that is corrected for variation in transfection efficiency between different transfections. Luciferase assays are only widely used in cell lysates but comparable dual reporter strategies have also been used in image-based assays (Bhatia et al., 2015).

A related issue is that it is not straightforward to predict candidate TFBSs to mutate in enhancer assays. *In vitro* binding assays do not necessarily reflect *in vivo* patterns of TF occupancy, and many *in vivo* binding sites are likely not functionally important (Yang et al., 2006; Hu et al., 2007; Biggin, 2011). The standard approach is to computationally predict TFBSs in CRMs of interest based on prior knowledge of transcription factor binding motifs. Binding motifs are thought to be relatively conserved across taxa (Nitta et al., 2015) and there are large databases of TF binding motifs from vertebrate and invertebrate model organisms (Fornes et al., 2019). These are usually derived from SELEX, Protein Binding Microarrays (PBMs) or related approaches for selecting optimal binding motifs *in vitro* (Tuerk and Gold, 1990; Berger and Bulyk, 2006; Riley et al., 2014), but may also incorporate inferences from ChIPseq about consensus sequences *in vivo* (Ghandi et al., 2014; Nitta et al., 2015). SELEXseq data is also available for many *Ciona* TFs (Nitta et al., 2019). Binding motifs vary widely between different TFs, but they often involve a core sequence that is invariant or near-invariant flanked by sequences that are more variable. There are no widely accepted best practices, however, for how to exploit binding motif data for TFBS prediction. Many ascidian papers are actually quite vague about how candidate TFBSs were identified. One strategy is to look for perfect matches to the core motif, but that depends on the core motif being absolutely invariant and discards potentially useful information in the flanking nucleotides. An alternate approach is to computationally scan for a probabilistic match to the entire binding motif, but this depends on a statistical threshold for determining what counts as a match that usually cannot be defined *a priori* in any principled way. There are also important details related to the scanning algorithms used and the different statistical frameworks such as Position Weight Matrices and k-mer tables that can be used to encode binding preferences (Stormo, 2000; Ghandi et al., 2014). Complicating these matters further, there is evidence that some enhancers may have been tuned by evolution to make use of suboptimal binding sequences as part of unavoidable tradeoffs between the strength and tissue-specificity of expression (Farley et al., 2015; Farley et al., 2016).

An important challenge for ascidian developmental systems biology is to develop cis-regulatory reporter assays that are more quantitative and more explicit in their assumptions. That is our goal here, using the proximal enhancer for the notochord transcription factor Brachyury (Bra) as a test case. Brachyury induction in both the primary A-line and secondary B-line notochord lineages has been extensively investigated and numerous upstream regulators are known (Miya and Nishida, 2003; Yagi et al., 2004; Hudson and Yasuo, 2006; Imai et al., 2006; Matsumoto et al., 2007; Farley et al., 2016; Veeman, 2018; Harder et al., 2021; Reeves et al., 2021). Several of these interactions are likely direct based on mutating predicted TFBSs in reporter assays (Corbo et al., 1998; Fujiwara et al., 1998; Takahashi et al., 1999; Yagi et al., 2004; Matsumoto et al., 2007; Farley et al., 2016; Reeves et al., 2021), but there has been no systematic attempt to predict and disrupt sites for all of these putative upstream regulators in the same quantitative framework.

## Results

### Approach

**FIGURE 1:**
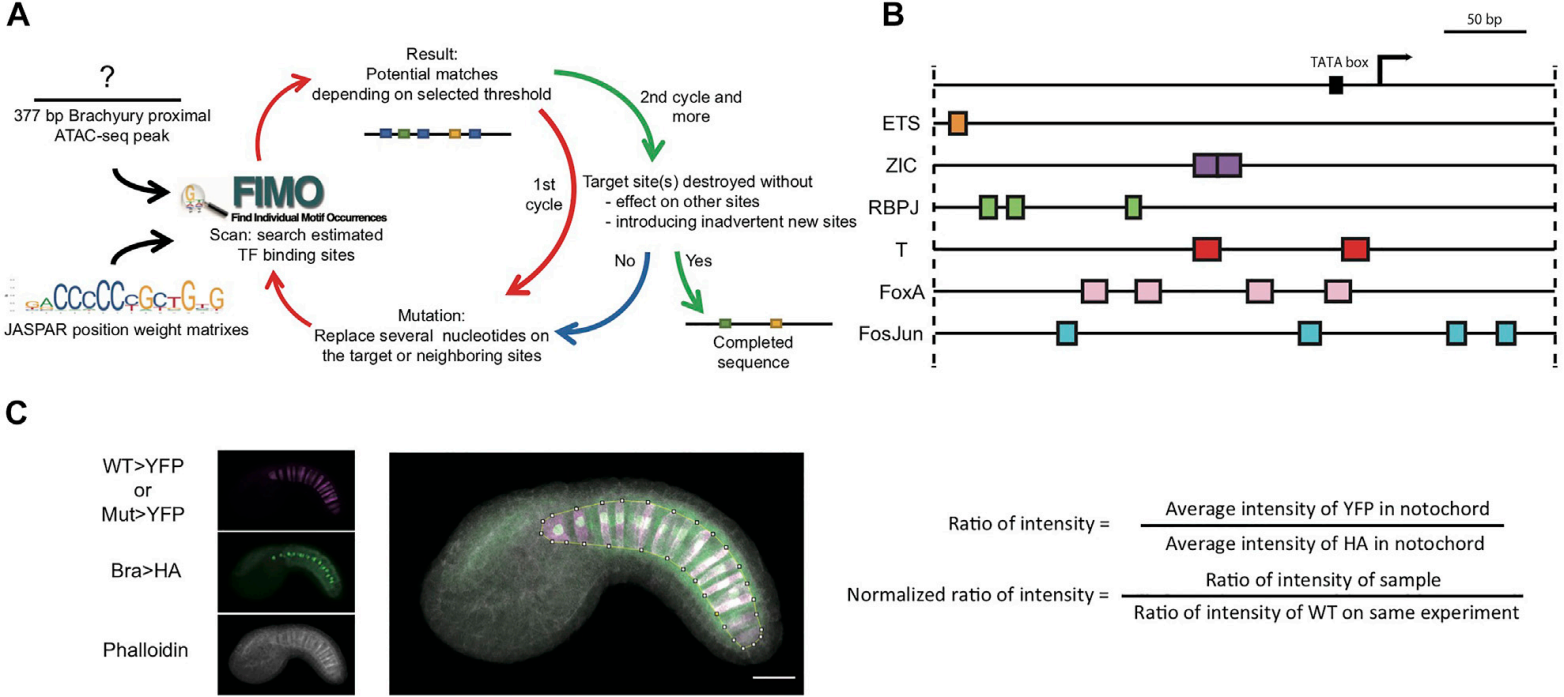
Systematic TFBS mutation and quantitative reporter analysis for the proximal brachyury enhancer. **(A)** Flowchart for TFBS prediction and mutation design. Black arrows, initial input. Red arrows, procedure for first cycle. Green and Blue arrow, second cycle and decision for completion of mutated sequence. **(B)** Diagram of predicted TFBSs on *bra* 377-bp promoter/enhancer. Arrow: endogenous *bra* transcription start site. The plasmid vector also contains its own bpFOG basal promoter. Colored boxes: predicted TFBSs. **(C)** Dual reporter image acquisition and analysis strategy. The YFP reporter channel is shown in magenta, and the Bra>H2B:HA internal control channel in green. Phalloidin staining is in greyscale. The overlayed projection is marked with a Region of Interest around the notochord.

We have developed a dual-reporter strategy for quantitatively dissecting the proximal *bra* enhancer (Figure 1). One plasmid contains a wildtype *bra* enhancer driving expression of an HA-tagged Histone H2B reporter as an internal control for electroporation efficiency. The other contains either a wildtype or mutated enhancer variant driving expression of YFP. We electroporate those together into fertilized *Ciona* eggs, fix embryos at stage 21, and immunostain for the two reporters. We clear the embryos in Murrays clear and then acquire high resolution confocal z-stacks through a sample of embryos. We quantify the results by sum-projecting the stacks to give a flattened 2D image, manually drawing a Region Of Interest (ROI) around the notochord and integrating the signal intensity for each reporter channel within that ROI. A normalized reporter value can then be calculated for each embryo by dividing the YFP intensity by the HA intensity. The results can then be further normalized to give the wildtype control a mean of 1.

We mutated predicted binding sites for 6 different TFs of interest. Zic, Ets and FoxA are all known upstream regulators of Bra (Yagi et al., 2004; Imai et al., 2006; Matsumoto et al., 2007; Farley et al., 2016; Reeves et al., 2021). Primary notochord cell fate specification depends on FGF signaling that culminates in the activation of Ets family TFs. Secondary notochord fate, in contrast, is thought to be induced by Notch/Delta signals that are transduced by Su(H)/RBPJ family TFs (Hudson and Yasuo, 2006). We looked at Bra sites [also known as T sites from the famous Bra mutation in mice (Herrmann et al., 1990)] to examine potential Bra autoregulation. We also looked at Fos/Jun (AP1) sites as there are several very strong matches in the proximal Bra enhancer.

For each of these TFs of interest, we searched the 377 bp *bra* proximal enhancer region using the FIMO scanner (Grant et al., 2011) and vertebrate PWMs from the JASPAR binding motif database (33). FIMO uses a statistical threshold expressed as a *p*-value. If the *p*-value is set too low, then few or no sites are identified. If it is set too high, then very large numbers of sites are identified that include very poor matches to the consensus sequence. We selected a *p*-value of 10^−3^ as an ad hoc compromise that produced a manageable list of relatively strong but not exact matches to the consensus motifs.

For each upstream factor of interest, we designed an enhancer variant in which all of the sites for that factor were mutated (Figures 1A, B). We then rescanned the variant sequence with FIMO to confirm that those sites were lost and to ensure that we had not inadvertently introduced other sites or affected overlapping sites. This sometimes required repeated cycles of variant design and FIMO scanning to find mutations that cleanly interfered with just the sites of interest. We also designed a construct in which the predicted sites for all six transcription factors were mutated simultaneously, and also three constructs containing combined mutations targeting two different upstream factors.

We had these variants synthesized as IDT gene blocks and then cloned them into a reporter plasmid containing the basal promoter from Friend Of GATA (bpFOG) and a YFP reporter. We co-electroporated each plasmid into fertilized *Ciona* eggs together with a longer wildtype *bra* enhancer construct driving Histone H2B:HA as an internal control for electroporation efficiency (Figure 1C). A typical experiment involved 4 or 5 electroporations, one of which always had the wildtype enhancer in both vectors as an additional control. Each variant was tested in at least three biological replicates in overlapping combinations with the other constructs.

### Initial Comparisons

**FIGURE 2:**
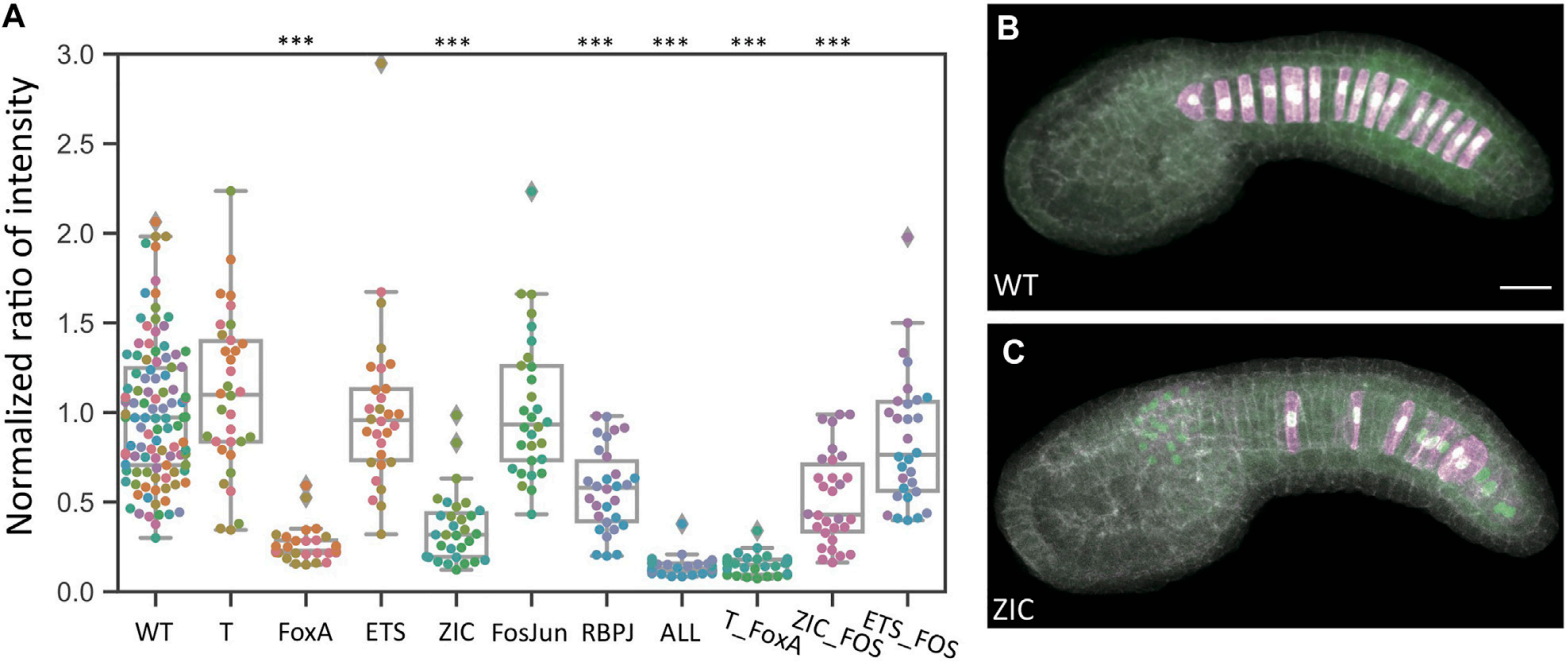
Quantitative effects of TFBS mutations in the *bra* proximal enhancer. **(A)** Comparison of normalized dual reporter intensity ratios for the wildtype and mutant *bra* reporter constructs. Each datapoint represents a different imaged embryo. Each color indicates embryos from the same replicate. The box plots indicate the outlier-adjusted minimum, 1st quartile, median, 3rd quartile and outlier-adjusted maximum for each construct. Outliers are marked as lozenges. Asterisks indicate differential reporter expression between the TFBS mutation construct and the wildtype construct. ***p < 0.001. (B–C) Representative sum-projected confocal images. **(B)**, wild type reporter construct. **(C)**, ZIC mutant reporter construct. Magenta: YFP reporter expression. Green: Bra>H2B:HA internal control expression. Gray, phalloidin. Scale bar, 20 µm.

Figure 2A shows an initial analysis of reporter intensity for all ten *bra* enhancer variants. Representative images are shown in Figures 2B,C. Each data point represents a different embryo imaged, and the reporter intensity values have been normalized to the internal control plasmid to correct for variable electroporation efficiency and then scaled to give the wildtype enhancer a mean value of 1. There is considerable variation in wildtype expression even after the dual reporter normalization, but most data points are between 0.5 and 2. Several constructs show a major decrease in reporter expression. The “ALL” construct in which every predicted site for all 6 TFs of interest was mutated showed the largest decrease. The FoxA and Zic mutations also led to major decreases. The RBPJ mutant construct had a smaller but still statistically significant decrease. The T, FosJun and Ets mutant constructs however showed no discernable effect. Supplementary Table S1 shows statistical tests for all pairwise comparisons between these constructs.

The three double mutant combinations tested did not reveal any major synergy. The T/FoxA mutant combination drove slightly weaker expression than the construct in which only FoxA sites were mutated, but this difference was not statistically significant. The Ets/FosJun mutant combination similarly drove slightly lower expression than the Ets or FosJun mutant constructs individually, but this was again not statistically significant. The Zic/FosJun mutant combination was similar to the Zic mutant construct.

**FIGURE 3:**
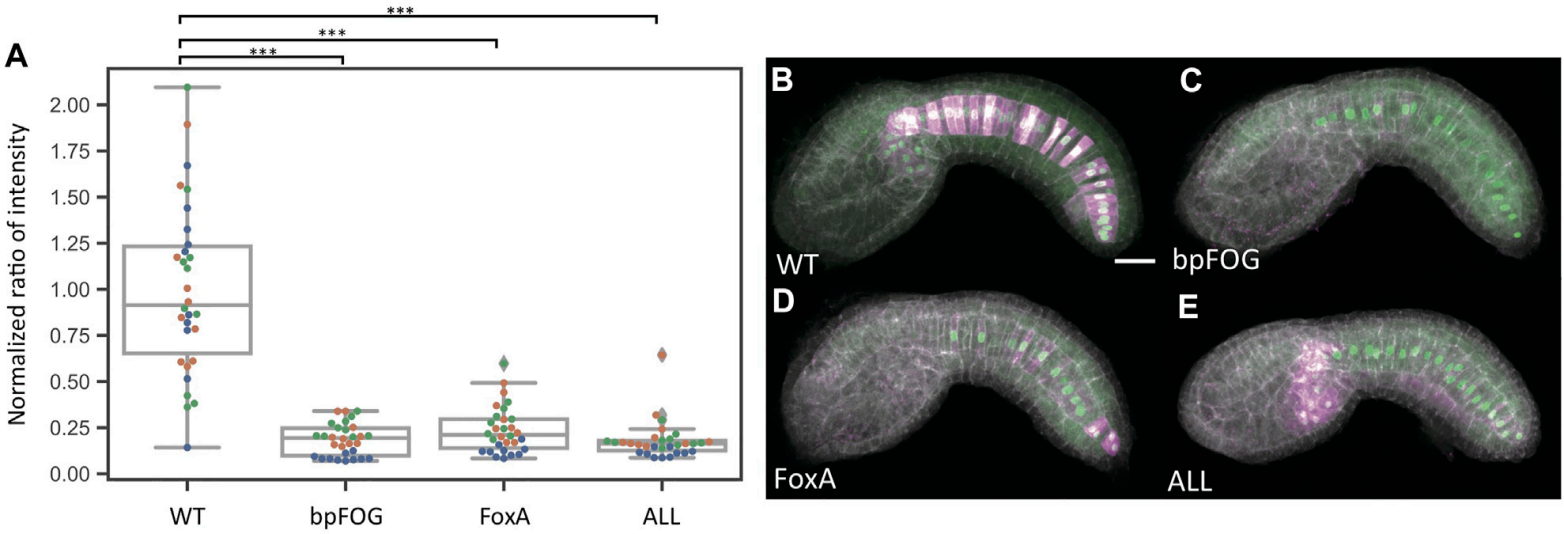
Baseline determination. **(A)** Normalized reporter expression for an empty vector containing only the bpFOG basal promoter and the two most severe mutant constructs. Each datapoint represents a different imaged embryo. Each color indicates embryos from the same replicate. The box plots indicate the outlier-adjusted minimum, 1st quartile, median, 3rd quartile and outlier-adjusted maximum for each construct. Outliers are marked as lozenges. Asterisks indicate differential reporter expression between the empty or mutant construct and the wildtype construct. ***p < 0.001. **(B–E)** Representative sum-projected confocal images. **(B)**, wild type. **(C)**, empty construct containing only the bpFOG basal promoter. **(D)**, FoxA. E, ALL. Magenta: YFP reporter expression. Green: Bra>H2B:HA internal control expression. Gray: phalloidin. Scale bar: 20 µm.

It was not clear if the very weak expression seen with the “ALL” construct and the FoxA mutant construct represented residual enhancer activity in these constructs or whether it might represent basal enhancer-independent expression from the minimal promoter (bpFOG) present in the dual reporter vectors. To test this, we performed additional reporter assays where we compared empty vector to the wildtype, FoxA mutant and “ALL” mutant constructs. Expression levels were comparable between the empty vector, FoxA and “ALL” constructs, indicating that the FoxA and “ALL” constructs lack any detectable enhancer activity (Figure 3 and Supplementary Table S2).

### Importance of Low Scoring ETS Sites

**FIGURE 4:**
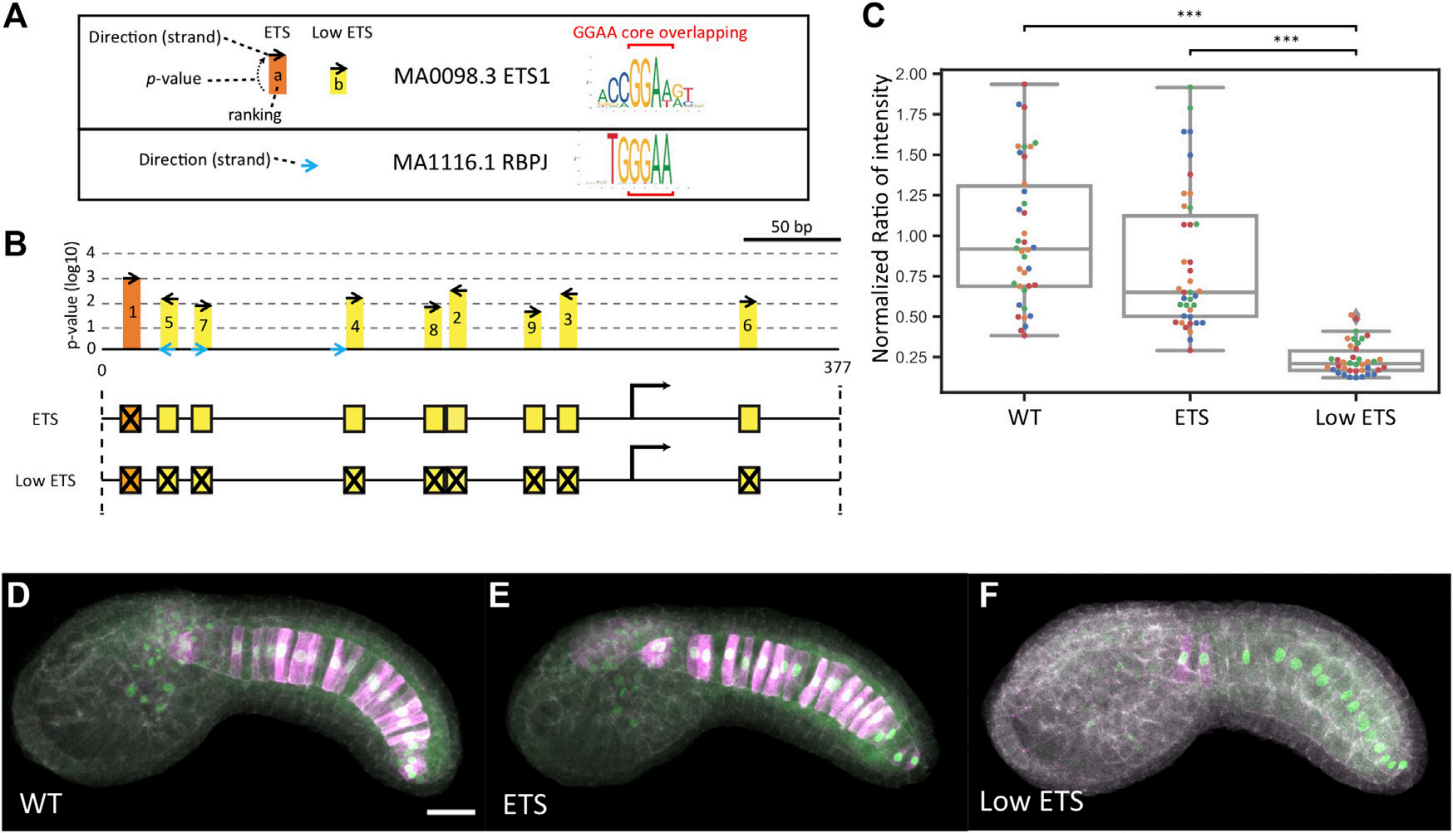
Analysis of low scoring ETS sites. **(A)** Sequence logos for the ETS and RBPJ Position Weight Matrices used in this study with the overlapping GGAA core indicated with red brackets. On the left is a key for the symbols used in the map in B). **(B)** Map of the *bra* proximal promoter/enhancer region showing the high scoring ETS site mutated in the initial ETS construct (orange) and 8 additional lower scoring sites mutated in the Low ETS construct (yellow) Putative RBPJ sites are shown in blue. **(C)** Normalized reporter expression for the ETS construct versus the Low ETS construct. Each datapoint represents a different imaged embryo. Each color indicates embryos from the same replicate. The box plots indicate the outlier-adjusted minimum, 1st quartile, median, 3rd quartile and outlier-adjusted maximum for each construct. Outliers are marked as lozenges. Asterisks indicate differential reporter expression between the mutant construct and the wildtype construct. ***p < 0.001. **(D–F)** Representative sum-projected confocal images. **(D)**, wild type. **(E)**, ETS. **(F)**, Low ETS. Magenta: YFP reporter expression. Green: Bra>H2B:HA internal control expression. Gray: phalloidin. Scale bar, 20 µm.

An immediate question was why the ETS mutant construct drove expression at the wildtype level despite it being well accepted that FGF signaling directly induces Bra expression. One possible explanation is that our *p*-value threshold for identifying ETS sites was too low and that we failed to mutate functionally important TFBSs. A challenge here is that the core binding motif for Ets family TFs is very similar to the core motif for RBPJ and sites for these factors may overlap (Figure 4A). We had initially theorized that a relatively stringent *p*-value threshold might allow us to cleanly separate between ETS-mediated and RBPJ-mediated expression even if we failed to mutate all potential sites. With our initial threshold of 10^−3^ we only identified a single ETS site in this enhancer and we were able to design mutations predicted by FIMO to have a large effect on ETS binding while not strongly interfering with RBPJ binding. There are several other potential ETS sites, however, that missed our *p*-value threshold (Figure 4B). We designed a follow-up “Low ETS” construct in which we mutated these predicted low affinity sites as well. For this construct we were unable to design mutations that affected the predicted Ets sites without also interfering with 2 of the 3 putative RBPJ sites (Figure 4B). This construct did show a major effect with reporter expression decreased to baseline levels (Figures 4C–F and Supplementary Table S3).

### The RBPJ Mutant Reporter Shows a Greater Decrease in Secondary Notochord

**FIGURE 5:**
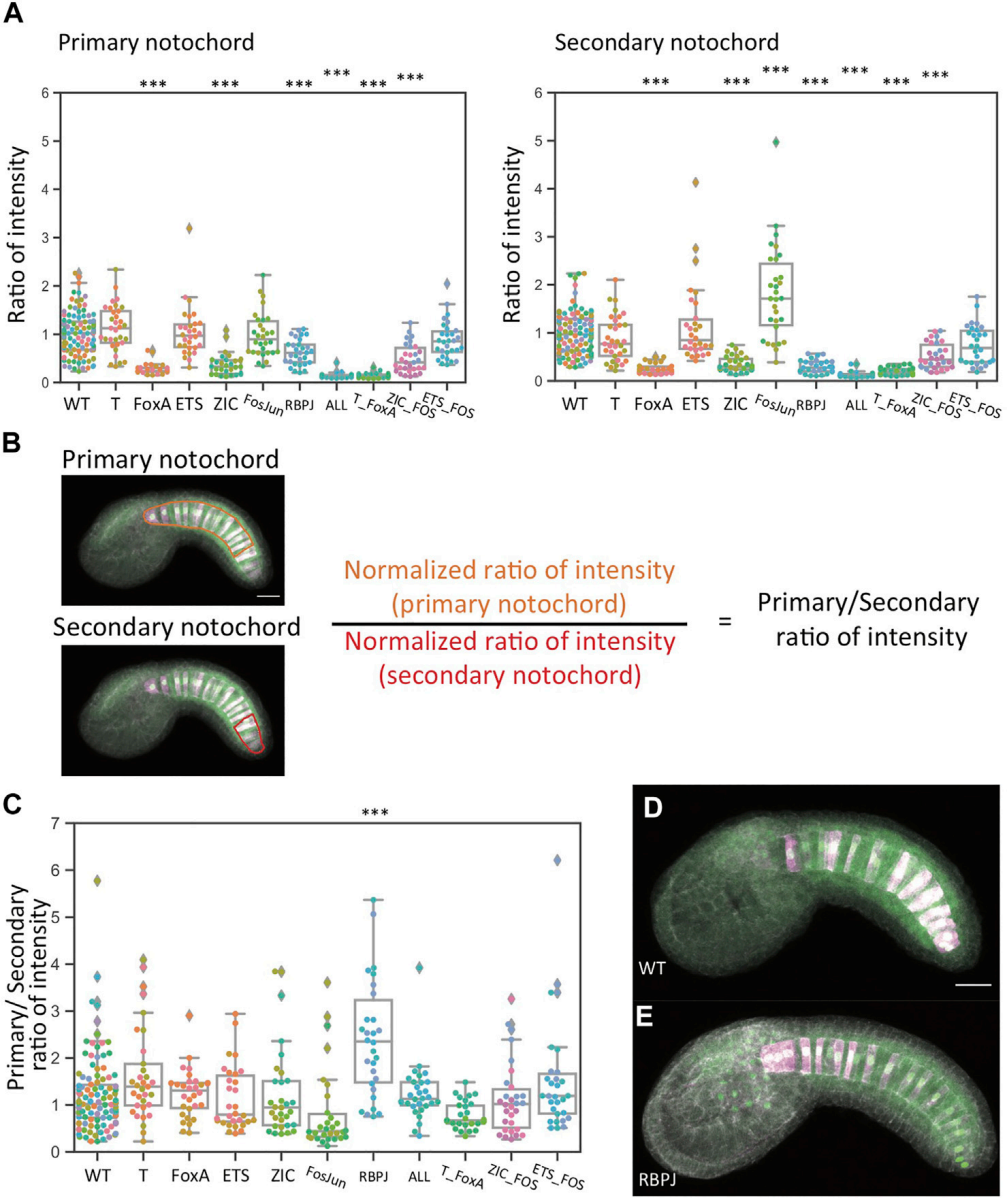
Analysis of reporter expression in primary versus secondary notochord. **(A)** Left: normalized reporter expression in primary notochord. Right: normalized reporter expression in secondary notochord. Asterisks indicate differential reporter expression between the mutant construct and the wildtype construct. ***p < 0.001. **(B)** Schematic indicating how the primary/secondary normalized expression ratio was calculated. **(C)** The ratio of normalized primary notochord reporter expression to normalized secondary notochord reporter expression. Asterisks indicate a differential primary/secondary ratio between the mutant construct and the wildtype construct. ***p < 0.001. **p < 0.01. *p < 0.05. **(D,E)** Representative sum-projected confocal images. **(D)**: wild type. **(E)**: RBPJ. Magenta: YFP reporter expression. Green: Bra>H2B: HA internal control expression. Gray: phalloidin. Scale bar, 20 µm.

The mechanisms of Brachyury induction are thought to be quite different between the primary A7.3/A7.7 derived notochord and the secondary B8.6 derived notochord (Yagi et al., 2004; Hudson and Yasuo, 2006; Imai et al., 2006). The sibling fate to primary notochord is the A-neural lineage, and the bifurcation between these cell states is known to be controlled by FGF/MAPK signaling. In the secondary notochord lineage, FGF signaling is thought to only be involved indirectly *via* the induction of b6.5 fate at the 32-cell which triggers a relay mechanism involving Nodal and Delta expression. The cell state bifurcation between B8.6 secondary notochord and its sibling B8.5 mesenchyme is thought to be proximately controlled by Delta/Notch signaling from A7.6 (Hudson and Yasuo, 2006). We did not note major overt differences in primary versus secondary expression with any of these constructs, but we speculated that quantitative analysis might reveal subtler effects. To test this, we reanalyzed the data using separate ROIs for the primary and secondary notochord. Figure 5A shows reporter expression in the primary and secondary notochord individually. Figures 5B,C shows the ratio of normalized primary expression to normalized secondary expression, which is centered around 1 for most constructs. Representative images are shown in Figures 5D,E. The major exception was the RBPJ construct, where the median ratio is above to 2. That indicates that expression in the secondary notochord was more severely affected by this set of mutations. This difference from wildtype was statistically significant. While not a complete loss in one lineage, this is the first demonstration of a differential effect on primary versus secondary expression for a mutant Bra enhancer variant. Supplementary Tables S4, S5 show statistical tests for all pairwise comparisons.

One unexpected observation is that the Fos/Jun mutant construct showed a statistically significant *increase* in expression in the secondary notochord. This implies that Bra expression in the secondary notochord may actually be under negative regulation *via* these sites. We speculate that this might involve unknown mechanisms that help balance Bra expression in the primary and secondary notochord despite it being induced by somewhat different upstream factors in these two lineages.

We also quantified expression in primary versus secondary notochord for our follow-up dataset comparing the initial Ets mutant construct to the Low ETS construct in which additional putative ETS sites were mutated (Supplementary Figure S1 and Supplementary Table S6). Normalized reporter expression in both primary and secondary notochord were reduced to near baseline levels for the Low ETS construct, but for the initial ETS mutant construct we now saw modest decreases in both primary and secondary notochord that were statistically significant in the primary lineage. A likely explanation for this discrepancy between the datasets is that the construct in which only a single putative ETS site is mutated leads to only a small effect on reporter expression and that our experiments lacked the statistical power to consistently detect this.

### Ectopic Expression

**FIGURE 6:**
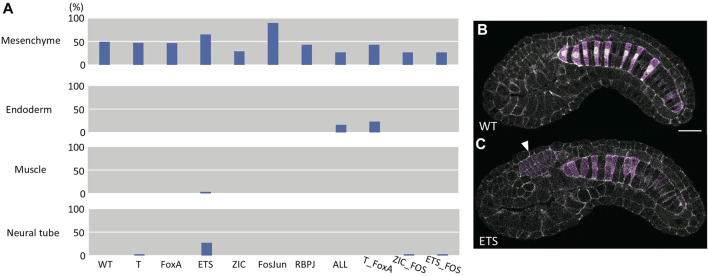
Analysis of ectopic expression. **(A)** Bar chart of the fraction of embryos scored as having ectopic reporter expression in the indicated tissues. **(B)** representative image of wildtype *Bra* reporter expression. **(C)** an example image of the ETS mutant reporter showing ectopic expression in the posterior ventrolateral neural tube (arrowhead). The images in B and C are single confocal sections. Magenta: YFP reporter expression. Green: Bra>H2B:HA internal control expression. Gray: phalloidin.

While our quantitative analysis of this large confocal dataset was restricted to the notochord, we also qualitatively scored each embryo for whether there was ectopic reporter expression in other tissues (Figures 6A–C). All the constructs showed considerable ectopic expression in mesenchyme, which is seen with the wildtype *bra* enhancer and many other *Ciona* enhancer constructs. The wildtype enhancer rarely drives expression outside of notochord and mesenchyme, and the same was true for most of the variants. We note that the original ETS mutant construct does show some ectopic expression in the neural tube, which is intriguing given that posterior ventral and lateral A-line neural tube is the sibling fate to primary notochord. We reassessed these image stacks to check whether the ectopic expression was regionalized and found that it did tend to be in posterior ventral and lateral neural tube consistent with it being from the A-lineage (Figure 6C). There was also increased ectopic expression of the Fos/Jun mutant construct in mesenchyme. We note that mesenchyme is in part the sibling fate of secondary notochord, which also showed increased expression with this construct.

### Single-Cell Reporter Quantitation

**FIGURE 7:**
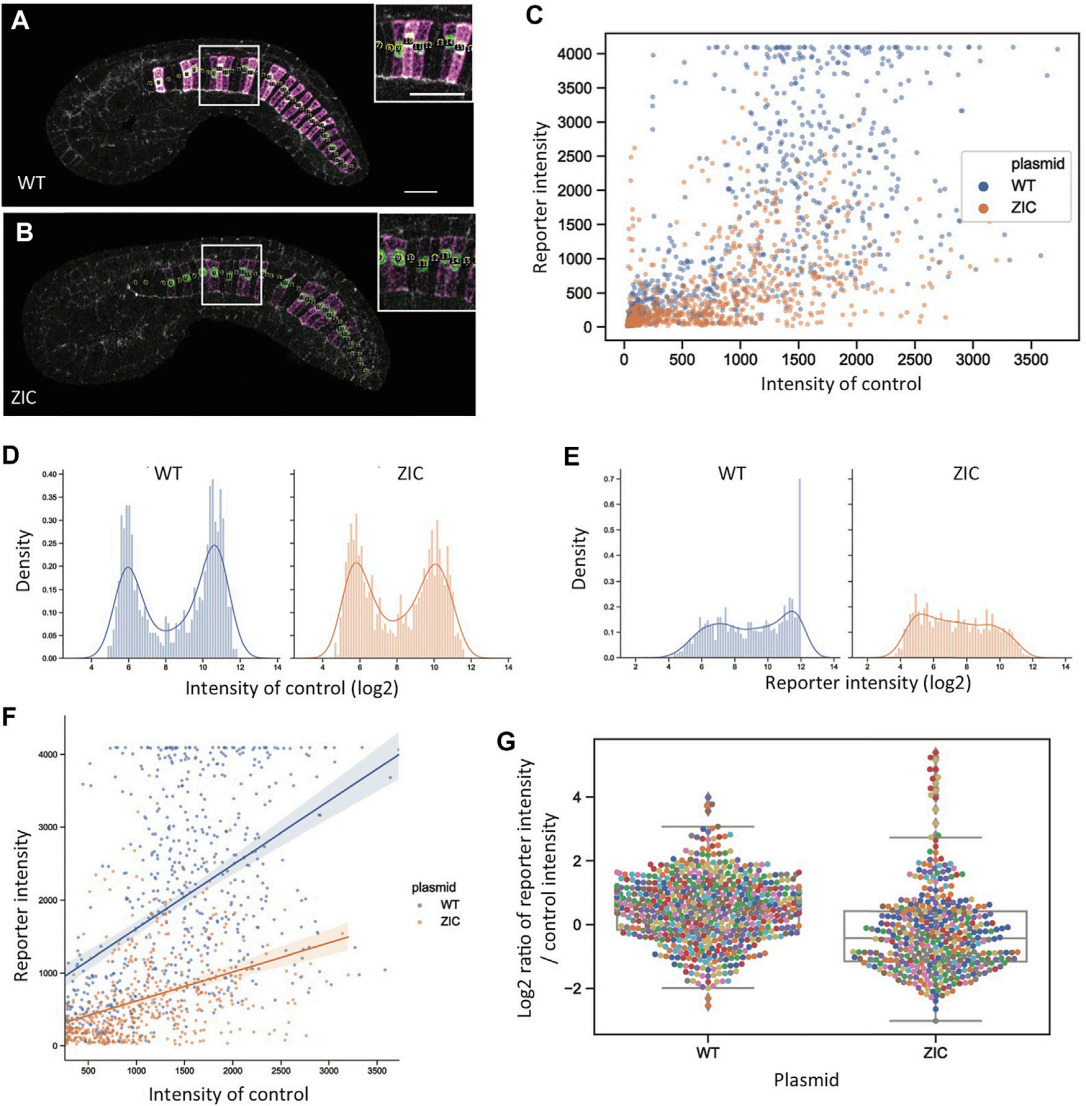
Single-cell reporter quantitation. **(A, B)** Computationally flattened mid-notochord slices for representative wildtype **(A)** and ZIC **(B)** embryos. Insets show zoomed in views with disk-shaped ROIs marked. Magenta: YFP reporter expression. Green: Bra>H2B:H internal control expression. Scale bar, 20 µm. **(C)** Scatter plot for intensity of reporter (*y*-axis) and internal control (*x*-axis). Blue: wildtype. Orange: ZIC. **(D,E)** Histogram distributions for the log2-transformed intensity of internal control **(D)** and reporter **(E)** expression. Blue: wildtype. Orange: ZIC. **(F)** Scatter plot of reporter and internal control intensity for cells with detectable internal control expression (internal control expression > 256). Linear regression lines show that there is still a relationship between reporter expression and internal control expression for the ZIC construct, but with a decreased slope compared to the wildtype construct. Blue: wildtype. Orange: ZIC. **(G)** Distributions of the log2-transformed ratio of intensities between reporter expression and internal control expression for the control expressing cells (internal control expression>256).

Reporter quantitation at the level of individual embryos is fast and straightforward but potentially obscures important details of reporter expression at the level of individual cells. For TFBS mutations that decrease but do not completely eliminate reporter expression, what is the nature of that decrease at the single-cell level? Are there fewer expressing cells but those cells express the reporter at wildtype levels (increased mosaicism) or is there a graded decrease in reporter expression at the level of individual cells? To examine this, we reanalyzed a subset of the confocal stacks to quantify reporter and internal control expression at the level of individual notochord cells (Figures 7A,B). We focused on the ZIC mutant construct and its matched wildtype controls as this construct showed a major but not complete loss of reporter expression.

Figure 7C shows a scatter plot of internal control and reporter expression for the wildtype reporter and the ZIC mutant construct. As expected for single cell measurements of gene expression, the data are quite noisy but it is clear that the ZIC mutant construct leads to a graded decrease in reporter expression and not just an increase in mosaicism. Cells that either do or do not express the internal control can be clearly distinguished based on the bimodal distribution of internal control expression (Figure 7D). The histogram distributions of reporter expression (Figure 7E) are less distinctly bimodal, likely reflecting the increased variance of the more diffuse YFP reporter compared to the concentrated nuclear signal of the H2B:HA reporter used for the internal control plasmid. While reporter expressing and non-expressing cells can’t be perfectly distinguished, there is a moderate increase for the ZIC construct in the fraction of cells with very weak reporter expression, consistent with a modest increase in mosaicism. Increase mosaicism is not, however, a sole explanation for decreased reporter expression. Figure 7F shows a scatterplot of reporter and internal control expression restricted to the cells expressing the internal control. There are a small number of ZIC mutant construct cells with strong internal control expression and effectively no reporter expression. Most cells, however, that express the internal control do express the reporter, but do so at considerably weaker levels. The same graded decrease can be seen looking at the ratio of reporter and internal control expression for the cells expressing the internal control (Figure 7G). We conclude that the loss of reporter expression in the ZIC TFBS mutant construct partially involves an increase in transgene mosaicism, but it also involves a graded decrease in reporter expression at the level of individual expressing cells. This is similar to what we previously observed quantifying the response of proximal and distal Bra enhancer reporter constructs to graded MAPK inhibition with intermediate doses of the MEK inhibitor U0126 (Harder et al., 2021).

## Discussion

### Cis-Regulatory Architecture of the Proximal *Bra* Enhancer

Several previous studies have mapped out cis-regulatory modules controlling *brachyury* expression in ascidian models (Corbo et al., 1998; Fujiwara et al., 1998; Takahashi et al., 1999; Yagi et al., 2004; Matsumoto et al., 2007; Farley et al., 2016; Harder et al., 2021; Reeves et al., 2021). Various transcription factor binding motifs of interest have been mutated, but these efforts have been split between *Halocynthia* and *Ciona* and also between the proximal *bra* enhancer and the more distal “shadow” *bra* enhancer in *Ciona*. This is the first attempt to disrupt predicted TFBSs for the full set of hypothesized direct upstream factors within a common experimental framework that allows quantitative comparisons between different mutant reporter constructs.

One somewhat unexpected finding was that our initial ETS mutant construct that destroyed the strongest predicted ETS site did not show a major decrease in expression when assessed in the quantitative dual reporter assay. We had previously assessed this construct in a less sophisticated quantitative reporter assay and seen a larger effect (Reeves et al., 2021). That previous effort lacked the internal control to quantitatively correct for electroporation efficiency and had fewer biological replicates, so we favor the conclusion here that this mutation does not cause a severe drop in reporter expression. The Low ETS construct in which additional sites of lower predicted affinity were mutated as well had a major drop in reporter expression, consistent with the expectation that FGF signals mediated by ETS family TFs are directly involved in Bra expression. The initial ETS mutant construct did show a modest phenotype in the second set of experiments where we compared it side by side to Low ETS, consistent with the idea that eliminating that single ETS site has a small but non-zero quantitative effect on primary notochord expression.

While we did see an intermediate effect with the RBPJ mutant construct, most of the constructs in which we perturbed predicted TFBSs for single upstream factors tended to have either very little reporter expression or else near-wild type reporter expression. It is possible, however, that subtler differences between these constructs may exist but that our experiments lacked the statistical power to detect them. A disadvantage of this approach is that collecting multichannel confocal stacks through stained and cleared embryos is far more time consuming than scoring X-Gal stained embryos by eye at the dissecting scope. In general, however, the results fit with our expectations that perturbations of Ets, Zic and FoxA sites should have major effects.

The lack of an effect from mutating predicted Brachyury (T) sites in this Bra enhancer was arguably unexpected. We had predicted this construct would have an expression defect given that tissue-specific cell fate regulators like Bra are commonly involved in autoregulatory positive feedback loops. It remains to be determined whether Bra is simply not involved in a direct autoregulatory loop, whether our experiments did not look for such an effect over the right time scale, or whether there may be additional important Bra (T) sites that we did not mutate.

Our quantitative analysis was most informative in looking at quantitative reporter expression differences between primary and secondary notochord. Extensive experimental evidence suggests that FGF signaling should be uniquely important for Bra expression in the primary notochord and Notch/Delta signaling in the secondary notochord. It is reasonable to expect that TFBS mutations affecting these lineage-specific upstream regulators should have lineage-specific effects. Prior efforts have not noted lineage specific differences with different *bra* enhancer mutations, but none of these earlier papers explicitly scored embryos for primary vs secondary expression. Here we found that the RBPJ mutant construct had decreased expression in both primary and secondary notochord, but the effect was distinctly more severe in secondary notochord.

One might also expect that ETS site mutations should lead to a stronger effect on primary notochord than secondary notochord, but that was less apparent. The Low ETS construct effectively eliminated reporter expression in both primary and secondary notochord. The initial ETS construct had no obvious phenotype in our first set of experiments and only a minor decrease in our second set of experiments. In that latter case, however, the effect was somewhat more prominent and only statistically significant in the primary notochord.

**FIGURE 8:**
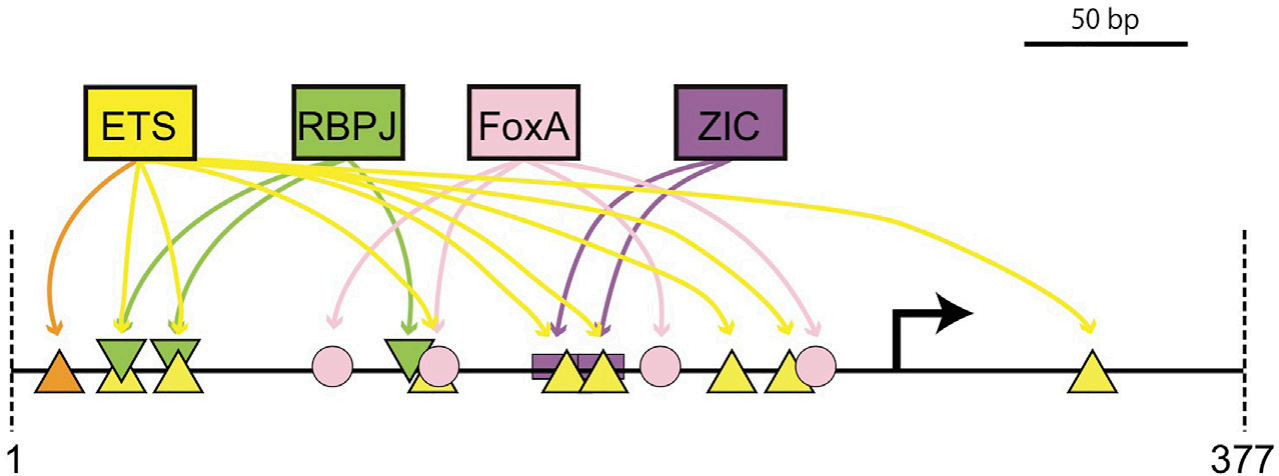
Cis-regulatory model for the proximal bra enhancer. Schematic diagram of major cis-regulatory inputs to the proximal *bra* enhancer showing the physical overlap between important predicted ETS and RBPJ sites. Orange and yellow triangles indicate high and low affinity ETS sites respectively.

There are several potential explanations for why expression in primary and secondary notochord were only partially separable in this study. The most obvious is that there is extensive physical overlap between predicted ETS and RBPJ sites. Using a match threshold of *p* < 10^−2^ for ETS and *p* < 10^−3^ for RBPJ, there are two positions where predicted ETS and RBPJ sites are completely overlapping. With less stringent thresholds, there would be more overlapping sites. We were able to design the RBPJ mutations in ways that were predicted to strongly interfere with RBPJ binding while having minimal effect on overlapping ETS consensus motifs, but it is plausible that the decrease in primary notochord expression with this construct involves overlapping low affinity ETS sites. Similarly, the complete loss of secondary expression with the Low ETS construct might reflect the loss of 2 of the 3 predicted RBPJ sites in that variant (Supplementary Table S7). It would be interesting to design new ETS and RBPJ constructs specifically targeting the sites with least potential overlap to see if primary and secondary notochord expression could be better separated. More broadly, this raises questions about potential crosstalk between MAPK and Notch/Delta signaling given the similarity in effector TF binding motifs. Figure 8 shows a summary model of the most important known cis-regulatory inputs to the proximal Bra enhancer that highlights the physical overlap between multiple ETS and RBPJ motifs.

An alternate possibility is that there may be more overlap than currently believed between the signaling pathways that induce Brachyury expression in primary versus secondary notochord. One possibility is that FGF signaling might have a direct role in the B8.6 vs. B8.5 cell fate decision in parallel to Delta/Notch signaling. This is difficult to test given that FGF signaling has earlier roles in inducing the B7.3 parental cell state (Kim and Nishida, 2001; Kim et al., 2007; Winkley et al., 2021) and also in triggering the Nodal/Delta relay by inducing b6.5 fate (Hudson and Yasuo, 2006). In our recent scRNAseq study we analyzed ETS site enrichment in the genes differentially expressed early in all the major cell fate decisions at the 64-cell, 112-cell and mid-gastrula stages (Winkley et al., 2021). We found that B8.6 vs. B8.5 had a very large ETS TFBS enrichment signature similar to several cell state bifurcations known to be directly controlled by FGF/MAPK signaling, suggesting that FGF signaling might be directly and not just indirectly involved. There are also hints to this effect in prior work including the observation of increased dpERK staining in B8.6 vs. B8.5 in some embryos, and the increased penetrance of the effect on secondary notochord fate upon morpholino knockdown of both FGF8/17/18 (late onset of expression) and FGF9/16/20 (early onset of expression) compared to FGF9/16/20 alone (Yasuo and Hudson, 2007). A further complication is that both scRNAseq and some in situs show that there is considerable Bra expression in B7.3 prior to secondary notochord fate restriction (Corbo et al., 1997; Winkley et al., 2021).

A third possibility is that these assays may be confounded by separable roles in the initiation and maintenance of Brachyury expression. One could imagine, for example, that the initial induction of Brachyury might depend on a combination of upstream ETS, RBPJ, Zic and FoxA factors but that later Bra expression might involve some sort of positive feedback loop. Our Bra(T) mutant construct did not have an obvious effect on reporter expression but it is possible we may have missed important Bra sites or the feedback loop might be indirect rather than direct. If there is a switch between different regulatory states for Brachyury induction and maintenance, it is possible that reporter expression driven by later maintenance factors could obscure early phenotypes when TFBSs for TFs involved in the earliest stages of Brachyury induction are mutated. It is possible that time series experiments with reporter assays performed at a range of different stages would lead to different interpretations than our single stage 21 endpoint.

### Quantitative cis-Regulatory Analysis

While these experiments were designed in part to learn more about the regulatory mechanisms controlling *Ciona* Bra expression, they were also intended as a proof of concept for a more quantitative and formalized approach to cis-regulatory analysis. Our initial hope was that a relatively stringent statistical threshold for TFBS prediction coupled with a very quantitative readout would allow functionally important TFBS families to be identified even if some potential low-affinity sites were missed. Our ad hoc choice of a FIMO p-val<10^−3^ against JASPAR PWMs proved adequate to identify functionally meaningful sets of Zic, FoxA and RBPJ sites, but a less stringent threshold was needed for ETS sites. This observation that ETS sites that are weak matches to consensus binding motifs are functionally quite important is consistent with the enhancer suboptimization hypothesis proposed by Farley and Levine (Farley et al., 2015; Farley et al., 2016).

Interestingly, both of the cases where Farley, Levine and colleagues explored the importance of suboptimal binding sites in the context of “enhancer grammar” also involved ETS sites, and one of their model enhancers was the more distal *bra* “shadow” enhancer (Farley et al., 2016). One interesting question is whether the use of suboptimal TFBSs is a characteristic feature of all enhancers and trans-acting factors, or whether there are consistent differences in the use of strong versus weak matches to the consensus binding motifs for different types of enhancer or different TF families. We speculate, for example, that the effector TFs regulated by signal transduction pathways might use suboptimal binding motifs more often than other TFs. This might involve enhancers that have been tuned by evolution to respond to precise thresholds of pathway activity. While there are no major morphogen gradients in *Ciona*, there are several cell types that depend on quite subtle quantitative differences in cell contacts between neighbors expressing agonists and antagonists of the Ets-mediated MAPK signals involved in many *Ciona* cell fate decisions (Tassy et al., 2006; Ohta et al., 2013). We note that mutation of strong matches alone was enough to give major phenotypes for the Zic, FoxA and RBPJ mutant constructs. We did not test for the potential importance of weaker matches for Fos/Jun or Bra (T), but there is no strong expectation from prior work that these sites should definitely be important. ETS sites were the only case where prior work strongly suggested they should be involved, but where the 10^−3^ threshold was inadequate to identify sites of major functional importance.

In the context of this special issue on invertebrate chordate systems biology, it is likely that cis-regulatory reporter assays will continue to be a mainstay of *Ciona* research and that these assays will become increasingly quantitative. Our efforts here are an attempt to begin developing a more quantitative framework for *Ciona* cis-regulatory assays, but there are many potential ways that quantitative enhancer assays could be implemented. They could use non-image-based readouts such as luciferase assays or deep sequencing. They could use other image-based strategies such as the direct imaging of two fluorescent protein variants for the reporter and internal control. Many distinct approaches for image analysis and reporter quantitation are possible and these methods could potentially be more extensively automated.

One major challenge is that the combinatorial space of enhancer variants needed to deeply dissect cis-regulatory codes is enormous. Massively parallel reporter assays promise to greatly increase throughput (Farley et al., 2015; Inoue and Ahituv, 2015; Hughes et al., 2018), but are subject to the same fundamental concerns about how best to predict potential TFBSs of interest. This involves a balance between different types of error. Regardless of the matching algorithm used and the details of how binding preferences are computationally encoded, a very stringent approach will minimize the chance of inadvertently mutating sites that are actually controlled by entirely different upstream factors but will increase the risk of missing important cognate sites. Too relaxed an approach will identify all the “real” binding sites but will greatly increase the chance of inadvertently mutating *other* important sites. This is complicated further by the fact that overlapping binding sites may often be of considerable biological importance. Best practices for TFBS prediction and systematic enhancer dissection are not currently clear, but are likely to emerge through the concerted efforts of the *Ciona* cis-regulatory research community.

## Materials and Methods

### *Ciona* Husbandry and Embryology

Adult *Ciona robusta* (formerly known as *Ciona intestinalis* type A) (Pennati et al., 2015) were collected in San Diego, shipped to KSU by Marine Research and Educational Products Inc. (M-REP, San Diego), and housed before use in a recirculating aquarium. Standard fertilization, dechorionation and electroporation protocols were used (Veeman et al., 2011). Staging is based upon the series of Hotta (Hotta et al., 2007).

### Preparation of Mutated *Bra* Enhancer Constructs

The wildtype proximal *Bra* enhancer used here is a 377 bp fragment derived from an ATAC-seq peak (Madgwick et al., 2019) over the upstream region and first exon of the *Bra* locus (KhS1404:5981-6357). The current *Ciona* peakome in ANISEED has been trimmed to remove the partial overlap with the transcribed region, but it was present when we designed this construct. This fragment was cloned into a vector containing a minimal promoter from *Ciona* Friend of Gata (bpFOG) and a Venus YFP reporter (pX2+bpFOG>UNC76:Venus (Stolfi et al., 2015)) as first described in (Reeves et al., 2021). Predicted transcription factor binding sites were identified using JASPAR 2018 PWMs and the FIMO scanner (Grant et al., 2011) using a *p*-value threshold of 0.001 for the initial study and 0.01 for the follow-up analysis of low-scoring ETS sites. Mutant sequences were designed to disrupt specific binding sites by altering important motif nucleotides to the other purine if it was a purine or the other pyrimidine if it was a pyrimidine. This ensured that both palindromic and non-palindromic binding motifs were disrupted. Most TFBSs were disrupted by mutating two to four nucleotides. This required care to avoid interfering with overlapping motifs, especially for longer motifs such as BRA and ZIC, and also core-sharing motifs such as ETS and RBPJ. For the RBPJ mutant we were able to interfere with highly conserved flanking residues without altering the GGAA core sequence. For the Low ETS construct, however, we were unable to mutate some ETS sites without disrupting overlapping predicted RBPJ sites. Full sequences of the wildtype and mutant enhancers are provided in Supplementary Data File S1. Supplementary Table S8 provides more details about each TFBS mutation.

All constructs were the same length and the spacing between different motifs was not altered. Mutated sequences were rescanned by FIMO to confirm the loss of the target motif and ensure that they did not affect nearby sites or inadvertently introduce new sites for our PWMs of interest. Multiple cycles of variant design and FIMO scanning were sometimes needed. Control and mutant sequences were all synthesized as IDT gBlocks and verified by Sanger sequencing after Gibson cloning into the bpFOG Venus YFP reporter vector. An initial analysis of the wildtype, ZIC, ETS and FoxA constructs using a simpler reporter assay lacking the internal control plasmid was published previously (Reeves et al., 2021).

### Electroporation, Immunostaining and Imaging

All constructs were electroporated in at least three different biological replicates at 50 micrograms each in 800 µL electroporations. 30 micrograms of Bra>Histone H2B:HA plasmid were included in each electroporation as an internal control for electroporation efficiency. This construct is from Harder et al. (2021) and contains 2.2 kB of *bra* upstream sequence spanning both the proximal and distal enhancers. Most individual experiments involved 4 or 5 different electroporations, one of which was always the 377-bp wildtype *bra* enhancer>Venus plasmid. All electroporations were performed as early as possible in the first cell cycle immediately after fertilization and dechorionation. All 4-5 electroporations in any one experiment were typically carried out within a 3–4 min window to minimize potential cell cycle effects on transgene mosaicism. For each experiment, embryos were fixed at stage 21, immunostained for reporter/internal control expression, and cleared in Murray’s Clear. For immunostaining, 1:1,000 anti-GFP (rabbit) (Fisher Cat. #A-11122) and 1:750 anti-HA (mouse) (Cell Signaling Cat. # 2367S) were used as 1° antibodies and with 1:1,000 anti-mouse Alexa Fluor 488 (Fisher Cat. #A11029) and1:1,000 anti-rabbit Alexa Fluor 555 (Fisher Cat. #A-21429) as 2° antibodies. The secondary antibody solution also included 1:150 Alexa 633 phalloidin (Fisher Cat. #A-12379)to label cell cortices. At least 9 embryos were imaged per construct per replicate using a 40×1.3NA objective on a Zeiss 880 confocal microscope using uniform imaging settings. Embryos which were well developed, had internal HA control signal in both primary and secondary notochord cells, and were uniformly oriented on their lateral side were arbitrarily selected for imaging without inspecting YFP reporter staining. The Z-stack range for imaging was set to include the entire notochord and lateral muscle tissues to ensure the notochord cells were completely imaged.

### Quantitative Reporter Analysis

We used a 2.5D analysis method in which we first Z-projected each stack in FIJI (Schindelin et al., 2012) using a sum intensity projection to flatten the stack into 2D without discarding intensity information along the *Z* axis. A polygonal Region of Interest was manually outlined around the notochord in each flattened image and used to quantify the mean image intensity within that ROI in both the reporter and internal control channels. For each embryo, we controlled for electroporation efficiency by dividing the reporter expression value by the internal control value. We further normalized the data on the level of each experiment to the mean ratio of the wildtype experimental plasmid. The analysis of primary versus secondary notochord expression was similar except that separate ROIs were made for the anterior 32 and posterior 8 notochord cells. The primary/secondary ratio for each embryo was calculated by dividing the reporter/control ratio in primary notochord by the reporter control ratio in secondary notochord. Analysis and visualization were performed using a combination of FIJI, Microsoft Excel and Python, including the Python Seaborn library. Statistical testing for differential reporter expression used the pairwise. t.test function in R with Benjamini-Hochberg adjustment to correct for multiple comparisons. The full tables of adjusted pairwise comparisons are provided in Supplementary Table S1–S6.

Data points from different replicate electroporations were typically intermingled in the distributions, indicating a general lack of major batch effects that were not corrected by the dual reporter normalization. There were occasional exceptions, however, such as the very low expression ratios for the mutant reporters from the replicate marked as blue in Figure 3. The nature of this batch effect is not understood, but it appears to be modest in scope. It might potentially involve subtle differences in embryo quality leading to threshold effects on the expression of weaker constructs.

### Evaluation of Ectopic Expression

Ectopic expression in mesenchyme, endoderm, muscle and neural tube were scored qualitatively by inspecting the projected images.

### Single-Cell Quantitation

Single cell measurements were made by first computationally reconstructing flattened slices through the middle of each notochord along its full AP length (Harder et al., 2019). We then manually selected disk-shaped ROIs of uniform radius that approximated the nucleus of each notochord cell and measured the mean intensity of the reporter and internal control channels in those ROIs using FIJI. While it lacks a nuclear localization signal and has more cytoplasmic staining that the H2B:HA reporter, the YFP reporter is quite nuclear (likely due to being excluded by cytoplasmic vesicles or lipid droplets) and is meaningfully captured by these ROIs. We separated control expressing and non-expressing cells using an internal control threshold of 256 (8 in log2 transformed values) based on the histograms in Figure 7D.

## Data Availability

The original contributions presented in the study are included in the article/Supplementary Material, further inquiries can be directed to the corresponding authors.

## Supplementary Material

Supplementary Figure S1 Separate analysis of ETS and low ETS constructs in the primary and secondary notochord. **(A)** primary notochord. **(B)** secondary notochord. **(C)** primary and secondary ratio of intensity. ****p* < 0.001. ***p* < 0.01.

Supplementary Table S1 Pairwise comparisons using t-tests with pooled SD for data of screening for mutation effect on major TFBSs predicted on Bra proximal enhancer. Red *p*-value < 0.05.

Supplementary Table S2 Pairwise comparisons using t-tests with pooled SD for data of determination of baseline and comparison between baseline and constructs with severe effect. Red *p*-value < 0.05.

Supplementary Table S3 Pairwise comparisons using t-tests with pooled SD for data of comparison of ETS and low ETS construct with wildtype. Red *p*-value < 0.05.

Supplementary Table S4 Pairwise comparisons using t-tests with pooled SD for data of separated analysis of mutation constructs with primary and secondary notochord. Above, primary notochord. Below, secondary notochord. Red *p*-value < 0.05.

Supplementary Table S5 Pairwise comparisons using t-tests with pooled SD for data of comparison of primary/secondary ratio of intensity. Red *p*-value < 0.05.

Supplementary Table S6 Pairwise comparisons using t-tests with pooled SD for data of Separated analysis of ETS and low ETS constructs with primary and secondary notochord. Above, primary notochord. Middle, secondary notochord. Below, primary/secondary ratio of intensity. Red *p*-value < 0.05.

Supplementary Table S7 The major difference between wildtype and Low ETS mutation constructs. Above to below, ETS, RBPJ, FOSB:JUN, FOXA1, ZIC1 and T sites. Left, wild type. Right, Low ETS. Gray, appropriately eliminated sites on Low ETS construct. Blue, the score was decreased but not completely eliminated sites on Low ETS construct. Pink, accidentally eliminated sites on Low ETS construct. Yellow, Pink, accidentally score increased sites on Low ETS construct.

Supplementary Table S8 Details of individual TFBS mutations.

Supplementary Data File S1 A multi fasta file of cloned bra enhancer sequences.

Supplementary Data File S2 FIMO result of each construct including wildtype and 11 mutated sequences. Orange, TFBS focused and mutated in this study. Blue, appropriately eliminated binding sites.

Supplementary Data File S3 Raw data of intensity and other measured values for the quantitative reporter assays

Supplementary Data File S4 Raw data of intensity and other measured values for the separate analysis of primary and secondary expression

Supplementary Data File S5 Raw data of intensity and other measured values for the reporter assay baseline experiments.

Supplementary Data File S6 Raw data of intensity and other measured values for the Low ETS experiments.

Supplementary Data File S7 Raw data of intensity and other measured values for the Low ETS experiments with separate primary and secondary notochord measurements.
