# Supplementary figures and images for: Quantitative Dissection of the Proximal Ciona brachyury Enhancer
Source: Front Cell Dev Biol. 2022 Jan 21;9:804032. doi: 10.3389/fcell.2021.804032 (PMC8814421; doi:10.3389/fcell.2021.804032)

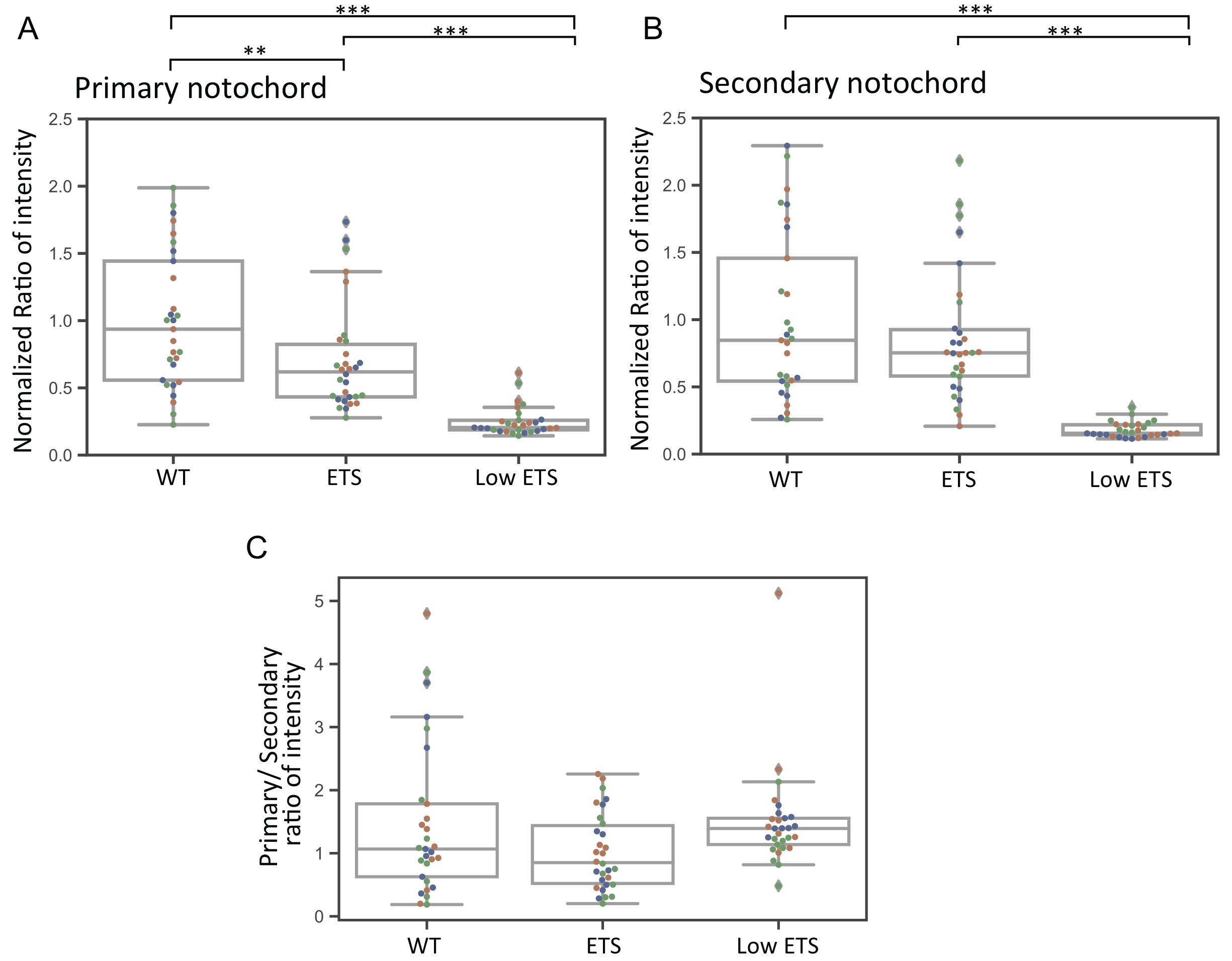

Supplement: Supplementary file 3 [file Image1.JPEG]
